# A long-term multisensor dataset from a coking plant soil lysimeter

**DOI:** 10.1016/j.dib.2025.112340

**Published:** 2025-11-28

**Authors:** A. Sobaga, N. Enjelvin, P. Faure-Catteloin

**Affiliations:** a Université de Lorraine, CNRS, LIEC, F-54000, Nancy, France; b Université de Lorraine, INRAe, LSE, F-54000, Nancy, France

**Keywords:** Soil water content, Soil matrix pressure, Soil temperature, Seepage, Weight, Soil profile

## Abstract

The datas come from a lysimeter column of 2 m of depth and 1m² of area located in the GISFI experimental station (Homécourt, France). This lysimeter was filled in 2008 by a coking plant soil contaminated with Polycyclic Aromatic Hydrocarbons (16 US-EPA PAH around 2000mg/kg), classified as a technosol (WRB classification).

Hourly measurements collected automatically using a datalogger, were performed at three different depths (50, 100 and 150 cm of depth): soil water content, soil matrix pressure and soil temperature. In addition, the total mass of the lysimeter and the seepage measured at the column bottom are also recorded. These measures are available from February 2008 until January 2025.

After data control, over 95 % of these measurement periods are considered reliable.

In addition, this dataset can be combined with data from a meteorological station localized in the same site to perform water budget.

All these measurements providing continuous, local and long-term observations, allow to study and forecast water transfers to an industrial technosol, for academic research, water agencies or citizens.

Specifications Table

**Table:** 

Subject	Earth & Environmental Sciences
Specific subject area	This dataset is part of the GISFI research consortium dedicated to the knowledge and the development of technologies for the requalification of degraded territories (http://www.gisfi.univ-lorraine.fr), and to have a better understanding of the evolution of water transfers and the recharge in anthropized soils.
Type of data	Table, Raw, Analysed, Unfiltered, Filtered,
Data collection	The lysimeter is a 2-meter-deep column with a surface of 1 m square. The column material is stainless inox316L. The soil used in the lysimeter originates from a former coking plant. It was excavated, sieved to 80 mm, and then placed into the column in successive layers of approximately 15 cm each. Description of soil characteristics is provided in the SoilMeasurement file. Data recorded every hour from 07 February 2008 to 31 December 2024. The variables measurement is soil temperature, soil water tension, soil water content at 50, 100 and 150 cm of depth. Seepage (S) is measured using a tipping counter at 2 m of depth and the total column weight (W) is also recorded. More details of the probes used are given in Table 1. This dataset is recorded at an hourly frequency and relayed to a datalogger and recorded in a local server in parallel on a cloud. Technical staff visit the site every week to check the equipment and data. Every two years, UGT, the company that designed and installed the system, inspects the installation and the probes. Further details are provided in the intervention file. Data has been filtered in order to obtain coherent chronicles. The Intervention file explains non-compliant (NC) data and missing acquisition (NA). In the final, over 95 % of these measurement periods are considered reliable.
Data source location	The data were collected in the GISFI station, Homécourt, France, (geographical coordinates : 49°21′ N, 5°99′ E; altitude 430 m), managed by the “Université de Lorraine”.In this region, the climate regime is continental. The weather station located on the site recorded a average annual temperature of 10.2 °C and a average annual precipitation of 727 mm for the period 2009–2024, with a well-defined temperature cycle. Database of the weather station are available between 2013 and 2017 in ORDAR: • https://doi.org/10.24396/ORDAR-127 • https://doi.org/10.24396/ORDAR-129 • https://doi.org/10.24396/ORDAR-142 • https://doi.org/10.24396/ORDAR-141.
Data accessibility	Repository name: ORDARData identification number: 10.24396/ORDAR-183 • Direct URL to data: Access to the Repository: https://ordar.otelo.univ-lorraine.fr/record?id=10.24396/ORDAR-183 • Intervention in the lysimeter: https://ordar.otelo.univ-lorraine.fr/files/ORDAR-183/Intervention_L05.csv • Soil Measurement: https://ordar.otelo.univ-lorraine.fr/files/ORDAR-183/Soil_Measurement_L05.csv • Unfiltered data: https://ordar.otelo.univ-lorraine.fr/files/ORDAR-183/data_lysi_L05_unfiltered.csv • Filtered data: https://ordar.otelo.univ-lorraine.fr/files/ORDAR-183/data_lysi_L05.csv
Related research article	None.

## 1 Value of the Data

• The data provides long-term and high frequency soil water dynamics in field condition at a pilot scale (2m^3^) for a technosol.
• This dataset is useful for understanding soil water dynamic at the interface between the atmosphere and groundwater.
• Researchers, public sector leaders, and population could use these data to assess the impact of climate change and land cover on water resources, in the past, present and future.
• These data provide the information needed to parameterize numerical hydrological models.
• Combining this dataset with quality monitoring data will provide better monitoring of contaminants (e.g. PAHs).

## 2 Background

In the context of climate change, information on water resources is needed to improve our understanding of the water cycle, particularly for monitoring groundwater recharge.

Lysimeters are the only instruments providing direct, local access to groundwater recharge [12,13]. Lysimeters are long-term devices that are mainly deployed on agricultural or forest soils for quality and quantity purposes [9].

This dataset gives an access to soil water dynamics in the soil and of the groundwater recharge, which is one of the twenty-three major challenges facing the hydrological community in the 21st century [4]. This dataset will enable to determine the water balance [2], and to evaluated the influence of a variation of land cover on the water budget in this region, and could be compared with other regions [1,11].

Technosols are still rarely monitored using lysimeters, even though they account for nearly 10 % of France's total land area. Although technosols are presented as promoting groundwater recharge in urban areas [6], they can evolve more rapidly than other soils and tend to become increasingly compacted [14]. In addition, the technosol used in this study (former coking plant soil) is contaminated by polycyclic aromatic hydrocarbons (PAHs), which can increase water retention. These datasets enable to fill these gaps with a technosol and could help assess water transfer and pollution in this specific soil.

## 3 Data Description

This article describes the set of data of one lysimeter localized in the GISFI station between 2008 and 2024.

There are four files in this dataset (Table 1): the file Soil_Measurement_L05.csv provides information on soil and physicochemical soil parameters. The file Intervention_L05.csv gives in detail the intervention on this lysimeter. Data_Lysi_L05_Unfiltered.csv contains unfiltered data and Data_Lysi_L05.csv, filtered data at hourly scale from 07 February 2008 to 31 December 2024.

**Table 1:** Content of the related dataset.

Files	Parameter Content
Soil_Measurement_L05.csv	Description of soil Horizon: Layers (-), Thickness (cm), Depth (cm), Weight (Kg), Bulk density (Kg/m^3^), Porosity (-). Physicochemical soil parameters: Soil Origin, Clay (g/kg), Fine silts (g/kg), Coarse silts (g/kg), Fine Sand (g/kg), Coarse Sand (g/kg), Total nitrogen (g/kg), CEC (meq/kg), Organic Carbon (g/kg), pH (-), Total limestone (g/kg), Cr (mg/kg), Cu (mg/kg), Ni (mg/kg), Zn (mg/kg), Co (mg/kg), Pb (mg/kg), Cd (mg/kg)
Intervention_L05.csv	The Intervention file explains NC non-compliant data and NA missing data. Interventions are identified by periods starting from start (from (YYYY-MM-DD) time (HH:MM:SS)) to end (to (YYYY-MM-DD) time (HH:MM:SS)). Some comments describe the intervention (Comments) and the measure affected (Measure affected).
Data_Lysi_L05.csv	Annual data recorded every hour from 07 February 2008 to 31 December 2024. date (YYYY-MM-DD), Time (HH:MM:SS), Seepage (S) (L), EQ_1 (kPa), Weight (kg), Soil Water volumetric content at 50, 100 and 150 cm (TDR_1, TDR_2, TDR_3, respectively) (%), Soil Temperature at 50, 100 and 150 cm (Temp_1, Temp_2, Temp_3, respectively) (°C), Soil Water tension at 50, 100 and 150 cm (EQ_1, Tens_2, Tens_3, respectively) (kPa).
Data_Lysi_L05_Unfiltered.csv	Annual data recorded every hour from 07 February 2008 to 31 December 2024. date (YYYY-MM-DD), Time (HH:MM:SS), Seepage (S) (L), EQ_1 (kPa), Weight (kg), Soil Water volumetric content at 50, 100 and 150 cm (TDR_1, TDR_2, TDR_3, respectively) (%), Soil Temperature at 50, 100 and 150 cm (Temp_1, Temp_2, Temp_3, respectively) (°C), Soil Water tension at 50, 100 and 150 cm (EQ_1, Tens_2, Tens_3, respectively) (kPa).

Soil water measurement of this dataset represented eleven parameters, and 1 625 261 data and give important information for scientists. Each variable is detailed in Table 2, including the measurement depth, the sensors employed, and their range and accuracy.

**Table 2:** Description of the soil measurement and probes associated.

Variable	Depth (cm)	Unit	Abbreviation	Sensor	Range	Accuracy
Soil Temperature	50- 100–150	°C	Temp_1 Temp_2; Temp_3;	UGT PT100 temperature sensor coupled with Tensio 300 EQ15 pressure sensor	−20 to +70 °C	±0.15 *à* ±0.3 °C
Soil Water Content	50–100–150	%	TDR_1; TDR_2; TDR_3	IMKO MICROMODULETECHNIK GMBH TRIME PICO32	0 to 100 %	± 1 % vol. H₂O for 0–40 % vol. ± 2 % vol. H₂O for 40–70 % vol.
Soil Water Tension	50	kPa	EQ_1	UGT Tensio 300 EQ15	+0 to −1500kPa	±0.2 kPa
Soil Water Tension	100–150	kPa	Tens_2; Tens_3	UGT Tensio 151	+20 to −85kP	± 0.3 kPa
Seepage	200	mm	S	Tipping counter VKWA100 100mL	–	± 0.1 mm
Weight	200	kg	Weight	SOEMER 3 Shearbeam Load Cell Model 3510	300 to 5000 kg	± 0.1 kg

For example, soil Temperature enable to monitor thermal conditions affecting soil, plant processes and biology activity, which can affect the carbon cycle [10]. Soil water content and soil water tension track the movement of water at different depth of the soil and enable to give information on the hydrodynamic parameters [16]. Seepage quantifies water flow through soil and provides information on groundwater recharge. Weight measures the variation in mass and therefore the storage of water in the lysimeter.

Abbreviation used in this dataset is constructed as follows: “*Parameter_z*” where Parameter could be “*Temp*” for Temperature, “*TDR”* for Time Domain Reflectometry; “*EQ”* for Equipotentiel, “*Tens”* for Tensiometer, “*S”* for Seepage, and “*Weight”* for the lysimeter column Weight. The suffix z (only for Temp, TDR, EQ and Tens) indicates the depth of the measurement (1, 2, 3 for 50, 100 and 150 cm, respectively).

These data are recorded and transmitted to a station computer directly via a datalogger. Periods with no data (NA) and inconsistent measurements (NC) are indicated in the datafile. In final, >95 % of these data are valid.

**Fig. 1:**
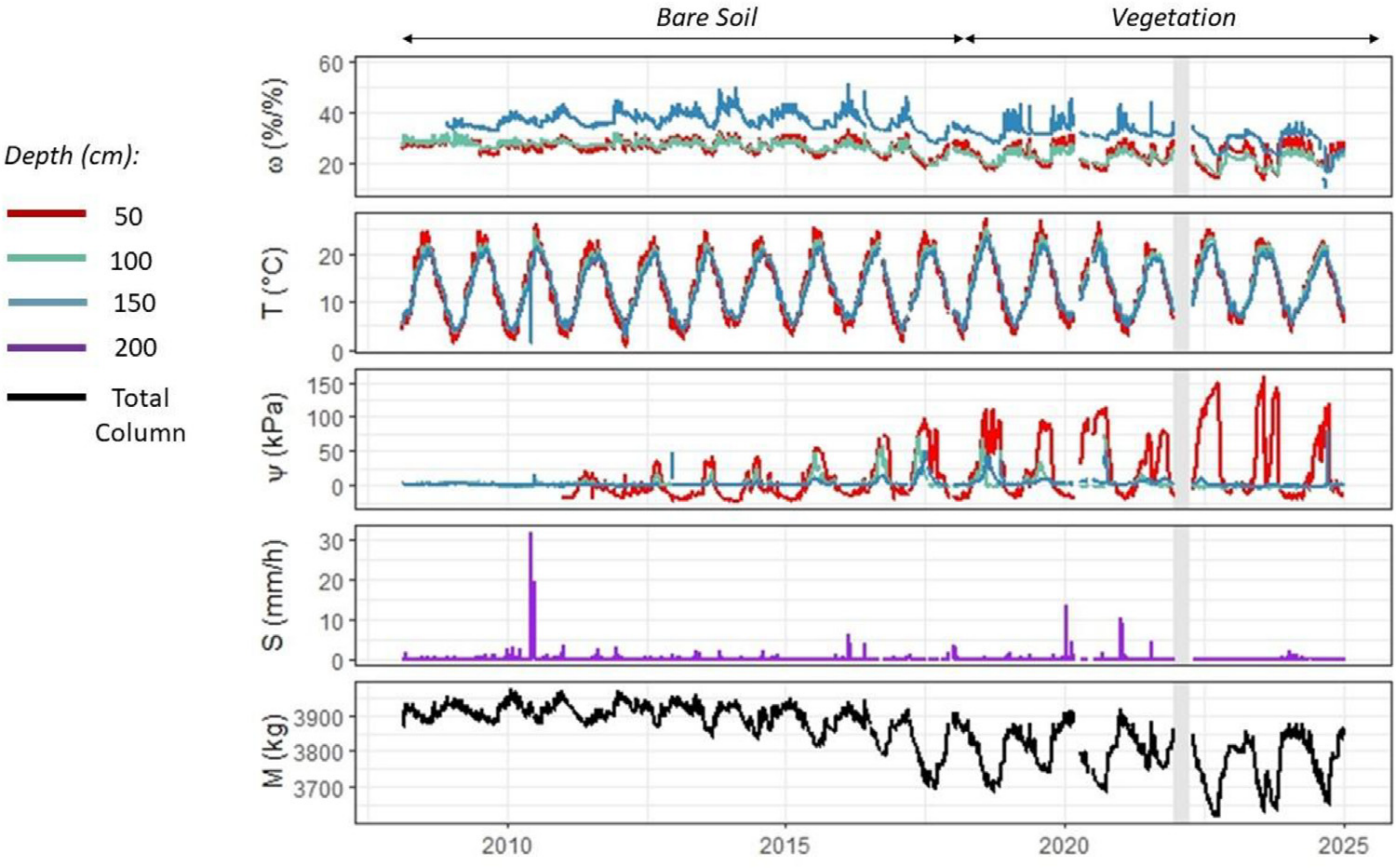
Hourly chronicles for the lysimeter: soil water content ω (%/%), soil water tension ψ (kPa) and soil temperature T (°C) at 50, 100 and 150 cm in yellow, green and blue respectively, the seepage S (mm/hour) at 200 cm and the weight of the lysimeter.

In the Fig. 1, hourly observation is represented from data filtered. Soil temperature exhibits a well-defined annual cycle, particularly at the surface (50 cm), with minimum values around 5 °C in February and maximum values of around 22 °C in July and August. In depth, the cycle is less pronounced (6 °C to 21 °C), and the maximum temperature is delayed by 1 month. Change in land cover do not influence soil temperature. Weight also exhibits a significant annual cycle (3877 to 3919 kg), with high values in winter and low values in summer (mininum in August). Changes in vegetation cover have significant impact with a more pronounced cycle due to the evapotranspiration from vegetation (3698 to 3862 kg).

In accordance with the weight, soil water content shows a similar pattern, with a more pronounced cycle at the surface (25 to 29 %/%) than at depth (26 to 28 %/%). Soil water tension shows a cycle that of soil water content, with high values in summer and low values in winter, particularly at the surface (−17 to 20 kPa). Annual cumulated seepage is 400 mm in bare soil and 260 mm in the presence of vegetation.

## 4 Experimental Design, Materials and Methods

### 4.1 Lysimeter design and filling methods

**Fig. 2:**
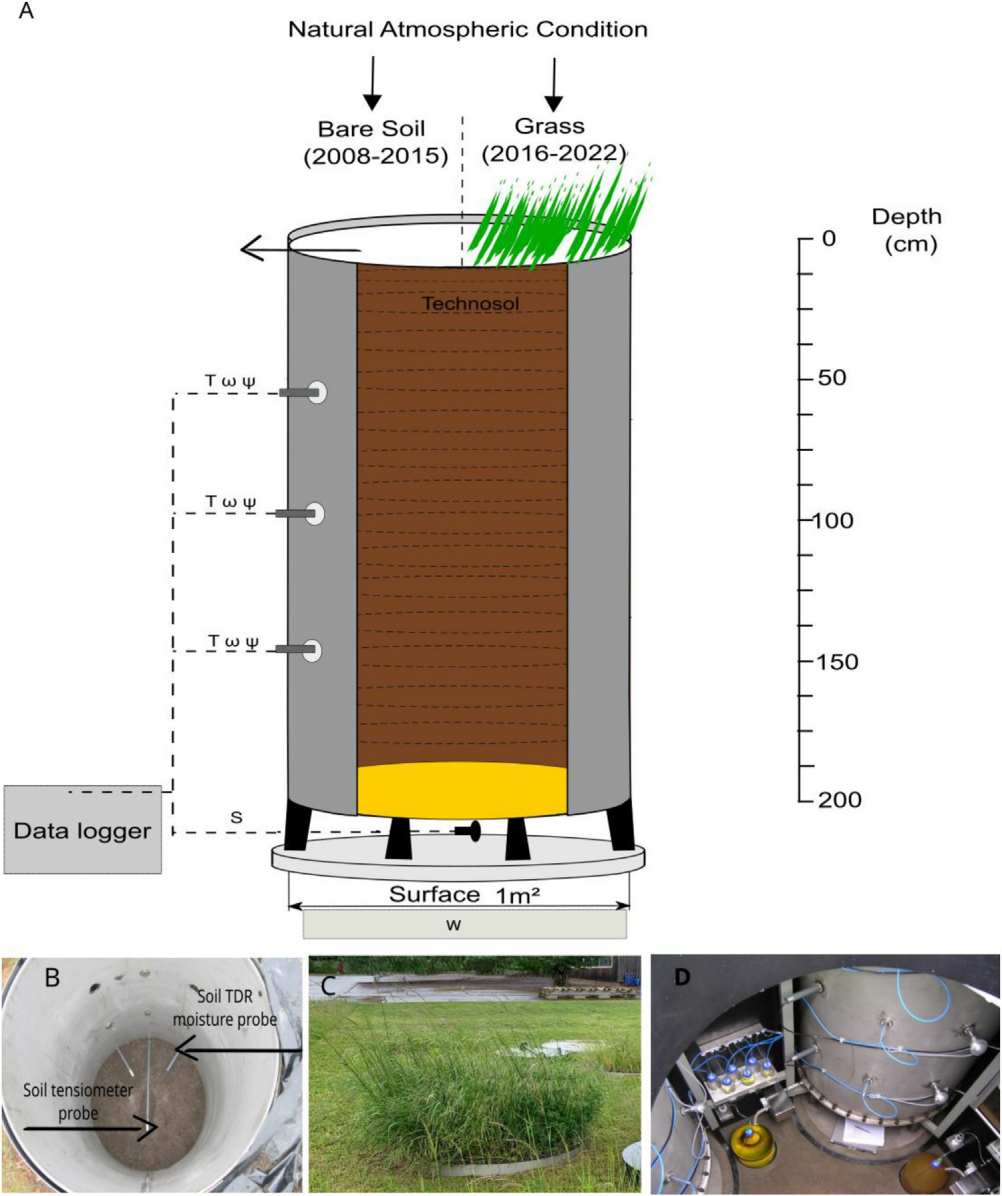
A. *Schematic diagram of the lysimeter used for data collection, illustrating key components involved in monitoring soil.* Measurement of Soil water Content (ω), Soil Matrix Pressure (Ψ) and Soil Temperature (T) at 50, 100 and 150 cm of depth. Seepage at 200 cm (S), and total weight of the column (W). B Lysimeter and probes - inside view. C. The vegetation present on the lysimeter on 29 may 2024. D. Lysimeter and probes - exterior view.

This lysimeter is a cylinder with a surface area of 1 m² and a depth of 2 m (Fig. 2A., B., C.). The original soil comes from the industrial wasteland of the town of Homécourt (Meurthe-et-Moselle, France), where a coking plant was located. This is a technosol, according to the WRB classification and polluted by coal-tars (including PAHs), metals [3,5,15].

More than 5 tons of soil was sampled during a campaign in July 2003. After homogenization and quartering, the soil was sieved at 15 cm and then at 4 cm. The soil was filled into the lysimeter according to the following protocol: a first layer of sand with progressive decrease in granulometry (3.0 – 5.6 mm, 0.7 – 1.0 mm, 0.1 – 0.5 mm) was placed at the bottom of the column to reach a thickens of 15 cm. Then the Technosol was manually deposited in the column by 10 cm (200 kg) layer and 100 tamper strokes of allowing reproducible controlled filling [7].

All the characterization of the soil is provided in the file ‘Soil_Measurement_L05.csv’ and were determined by the ‘Laboratoire d'Analyse des Sols d'Arras’. The soil studied is predominantly sandy. The pH of this soil is relatively high due to the limestone concentration. This soil has a high level of (i) organic carbon due to the occurrence of coal tar and (ii) metals, especially Cu, Zn, Pb and Cd, revealing a mixed pollution.

### 4.2 Soil–vegetation characteristics

Soil moisture parameters are estimated with soil moisture characteristic at 50, 100 and 150 cm depth, based on data collected between January 1 and June 31, 2013 (Table 3 Soil Hydraulic Parameters Table 3). The fit curve is based on the Van Genuchten equation [16], with the statistical pore-size distribution model of [8]. Assumption is done with θr is 0. The fitted parameters and regression statistics for the same period are presented in Table 1 for the three measurement depths (50, 100, and 150 cm). A proxy of the hydraulic conductivity at saturation (Ksat) could be estimated by the quantile 99 of the seepage, which is 1.6 mm/h (0.4 × 10⁻⁶ m/s).

**Table 3:** Soil Hydraulic Parameters.

Depth (cm)	$\theta_{sat}$	n	α	Number	AIC	R²	RMSE	MAE
50	*32*	1,13	0,02	278	−314,19	0,93	0,13	0,10
100	30,68	1,18	0,61	167	−236,67	0,98	0,11	0,07
150	42,2	1,13	1,76	163	−24.29	0,99	0,22	0,19

Until 2016, the vegetation is harvested regularly (Bare Soil). After 2016, the vegetation is not removed (Fig. 2B). The vegetation shown is spontaneous and consists of goldenrod (*Solidago*), knapweed (*Centaurea*), evening primrose (*Oenothera*), cinquefoil (*Potentilla*) and tansy (*Tanacetum*).The vegetation reaches a height of 0.5 m and extends over the entire surface (Fig. 2). There is no direct measurement of root depth because the system is closed; however, root depth can be estimated via its influence on soil water content through matric pressure (Table 4).

**Table 4:** Root depth in the lysimeter studied deduced by matric pressure measurement.

Periods	2018 - 2021	2022 - 2024
Root depth (cm)	100	∼150

### 4.3 Procedure for removing outliers

**Fig. 3:**
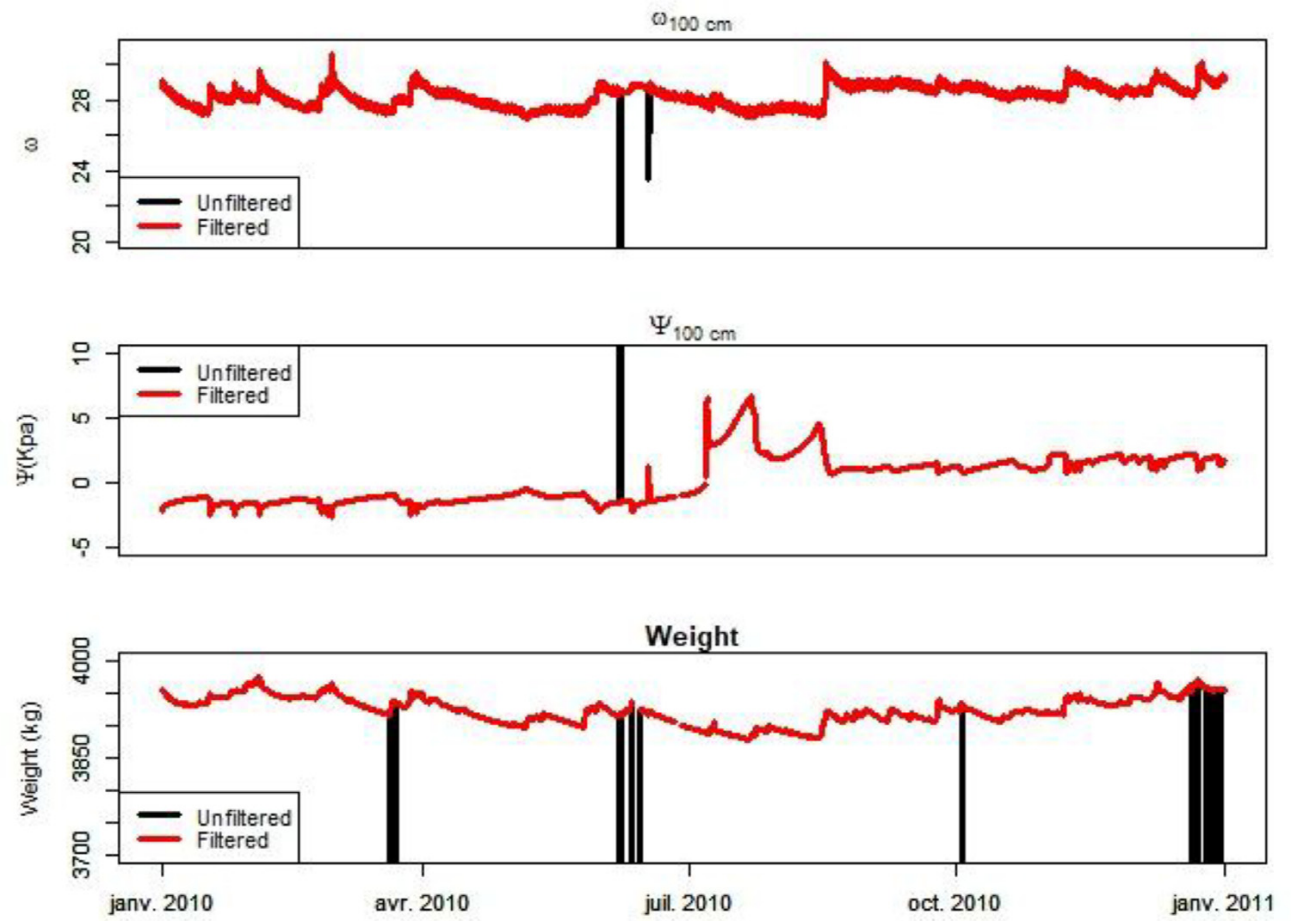
Comparison between Unfiltered (in black) and Filtered data (in red) for the 2010 chronicle.

An illustration is provided from 2010 chronicle between unfiltered (in black) and filtered (in red) data in the Fig. 3.

In final, >95 % of these data are valid over the entire period.

The procedure for removing outliers consists of excluding the following data:

• For Weight: values inferior to 500 kg.
• For seepage: negative values and values superior to 50 mm/h.
• For Soil Water Tension: values inferior to −30 kPa and superior to 80 kPa; values not included in the mean +−2 standard deviations; and hourly variation superior to |10| kPa.
• For Soil Water Content: values inferior to 10 %/% and superior to 80 %/%; and hourly variation superior to |5| %/%.
• For Temperature: not useful.

## Limitations

There are two main periods with no data, corresponding to the periods of Covid confinement and technical problems.

A curation step was performed for data identified as non-conform and defined in the datafile as NC. In the case of the soil tensiometers in the surface (50 cm), the data are only available after 2011.

## Ethics Statement

None

## Credit Author Statement

**A. Sobaga:** Conceptualization, Methodology, Software, Validation, Formal analysis, Investigation, Resources, Data Curation, Writing - Original Draft, Writing - Review & Editing, Visualization. **N. Enjelvin:** Conceptualization, Methodology, Investigation, Resources, Writing - Review & Editing, Supervision. **P. Faure-Catteloin:** Conceptualization, Methodology, Investigation, Resources, Writing - Review & Editing, Supervision, Project administration, Funding acquisition

## Data Availability

ORDARLysimeter L05 station GISFI Homecourt (Original data).
